# ICC-CLASS: isotopically-coded cleavable crosslinking analysis software suite

**DOI:** 10.1186/1471-2105-11-64

**Published:** 2010-01-28

**Authors:** Evgeniy V Petrotchenko, Christoph H Borchers

**Affiliations:** 1 University of Victoria - Genome British Columbia Protein Centre, Department of Biochemistry & Microbiology, University of Victoria, #3101-4464 Markham Street, Vancouver Island Technology Park Victoria, BC V8Z7X8, Canada

## Abstract

**Background:**

Successful application of crosslinking combined with mass spectrometry for studying proteins and protein complexes requires specifically-designed crosslinking reagents, experimental techniques, and data analysis software. Using isotopically-coded ("heavy and light") versions of the crosslinker and cleavable crosslinking reagents is analytically advantageous for mass spectrometric applications and provides a "handle" that can be used to distinguish crosslinked peptides of different types, and to increase the confidence of the identification of the crosslinks.

**Results:**

Here, we describe a program suite designed for the analysis of mass spectrometric data obtained with isotopically-coded *cleavable *crosslinkers. The suite contains three programs called: DX, DXDX, and DXMSMS. DX searches the mass spectra for the presence of ion signal doublets resulting from the light and heavy isotopic forms of the isotopically-coded crosslinking reagent used. DXDX searches for possible mass matches between cleaved and uncleaved isotopically-coded crosslinks based on the established chemistry of the cleavage reaction for a given crosslinking reagent. DXMSMS assigns the crosslinks to the known protein sequences, based on the isotopically-coded and un-coded MS/MS fragmentation data of uncleaved and cleaved peptide crosslinks.

**Conclusion:**

The combination of these three programs, which are tailored to the analytical features of the specific isotopically-coded cleavable crosslinking reagents used, represents a powerful software tool for automated high-accuracy peptide crosslink identification. See: http://www.creativemolecules.com/CM_Software.htm

## Background

Recent developments in the mass spectrometric analysis of proteins and peptides have led to renewed interest in the application of classical protein chemistry methods for structural elucidation of proteins and protein complexes. Advances in modern proteomics approaches, instrumentation, and methods for studying proteins and protein complexes structures has resulted in the development of the distinct field of structural proteomics. One of the most powerful methods in the structural proteomics toolbox is chemical crosslinking combined with mass spectrometry [1]. The idea behind this combination of techniques is straightforward -- to covalently modify proteins with reagents containing two reactive groups - *i.e*., crosslinkers -- to identify the sites of crosslinking and to deduce structural information about the protein system based on the spatial constraints derived from the length of the crosslinking reagents used. The current experimental paradigm is to enzymatically digest or to otherwise fragment these crosslinked proteins, and then to identify the crosslinked peptides (crosslinks) by mass spectrometry, thus determining the sites of crosslinking and providing information about protein conformation and protein-protein interaction sites. This approach inevitably creates a complex mixture of peptides in which unambiguous identification of the crosslinks is difficult.

One of the solutions to this problem is to use isotopically-coded crosslinking reagents [2]. When a crosslinker contains a mixture of chemically-identical light and heavy isomers, crosslinked products show up as doublets of peaks in the mass spectra. This provides a unique mass spectrometric "signature" for the detection of the crosslinks. Unambiguous assignment of the crosslinks based on their mass and MS/MS fragmentation patterns is another problem, due to the combinatorial possibilities of the constituents of inter-peptide crosslinks and the complexity of the MS/MS spectra because of the simultaneous fragmentation of two peptides per crosslink. To address this challenge, cleavable crosslinking reagents have been proposed [3]. Cleavage of the crosslinker converts the mass spectrometric analysis of a crosslink into the well-established analysis of the single peptides from which the inter-peptide crosslink was formed. Sequence data from the peptides making up the crosslink provides confirmation of the identity of these peptides and reduces the possibility of incorrect assignment of the crosslinks.

Particularly rewarding is a combination of these two features: isotopic coding and cleavage of the crosslinks [4]. Cleavage of the isotopically-coded crosslinks creates a new distinct signature for the cleaved crosslinks because the resulting pair of peptides might contain only a portion of the total number of isotopes in the uncleaved crosslinks. For example, the cleavage of the crosslinker BiPS, which contains eight deuterium atoms, leads to two peptides from each inter-peptide crosslink. Both of these peptides contain residual portions of the crosslinker, with each portion containing four deuterium atoms. Thus, the uncleaved crosslink will appear in the MS spectrum as a doublet of peaks 8 Da apart while the cleaved crosslink products will appear as doublets of peaks 4 Da apart. Knowledge of the cleavage reaction chemistry for each cleavable crosslinking reagent allows one to establish the specific mass relationships between uncleaved and cleaved crosslink signals which can be used as diagnostic crosslink-identification tools. Furthermore, crosslink assignments can be unambiguously confirmed by MS/MS analysis of both the uncleaved and the corresponding cleaved crosslinks.

Because of the large amount of data which is typically produced in the course of mass spectrometric analysis of crosslinked proteins, data analysis needs to be automated to make this analytical strategy feasible and applicable. Several software products have been proposed for this purpose [5-13], reviewed in [14]. The simplest approach described is to match the mass of a crosslink to possible combinations of the individual peptides predicted from protein sequences [5-7]. The success of this analysis depends both on the simplicity of the protein system and the mass accuracy of the MS measurements. The next level of confidence in assignment is achieved by programs taking into account MS/MS fragmentation of the crosslinks [8-11]. The discrimination of crosslinker-containing and non-containing fragment masses, distinguished from one another by the isotopic coding of the crosslinker, is the next step towards improving the efficiency and the accuracy of the crosslink identification process [12,13]. Unfortunately, straightforward use of the fragment ion masses is still sometimes not sufficient for unambiguous identification of the crosslinks derived from complex protein systems or whole proteomes [14].

To enable more confident and correct assignment of inter-peptide crosslinks, we have incorporated cleavage information in combination with isotopic coding into this new crosslink analysis software suite. We have developed a set of programs specifically designed for each step of an experiment where isotopically-coded cleavable crosslinking reagents are used. These steps are: 1) detection of the uncleaved and cleaved crosslinks (DX), 2) cleavage and identification of the cleavage products (DXDX), and 3) MS/MS fragmentation analysis of uncleaved and cleaved crosslinks (DXMSMS). We call this set of programs "ICC-CLASS" (Isotopically-Coded Cleavable CrossLinking Analysis Software Suite). Detection of the signals from the isotopically-coded crosslinks in the mass spectra based their isotopic signatures (the DX program) is done by searching the data for pairs of peaks with a mass increment corresponding to the mass difference between the heavy and light isotopic forms of the crosslinker. The DXDX program is designed specifically to provide automated isotopically-coded cleavable crosslink type identification based on cleavage information. DXMSMS program features separate input for isotopically-coded and non-coded fragment masses, input for possible cleavage products, as well as output of fragment masses for the cleaved crosslinks. Incorporation of crosslink cleavage data into the analysis greatly enhances confidence of crosslinks assignments. Here we describe in detail the functions and algorithms used in each module, as well as the overall structure of this software suite.

## Implementation

Programs were written in Microsoft Visual Basic 2008 Express Edition. Downloadable files are posted on http://www.proteincentre.com and the http://www.creativemolecules.com website at http://www.creativemolecules.com/CM_Software.htm. Executing the downloaded programs requires installed Microsoft .NET Framework, which is freely available from http://www.microsoft.com.

These programs are primarily oriented towards Applied Biosystems (AB) MALDI-TOF/TOF data from HPLC fractions, but can be used with any tab-delimited mass lists and are therefore instrument independent. We also have provided the text of these macros, along with installation instructions for instruments using AB's Data Explorer software, which automatically generates mass lists and lists of ion signal doublets from multiple MALDI-MS spectra. All results are saved in text files as tab-delimited values and can therefore be easily copied into Excel spreadsheets.

## Results and Discussion

### Analysis of the data from experiments using isotopically-coded cleavable crosslinkers

**Figure 1:**
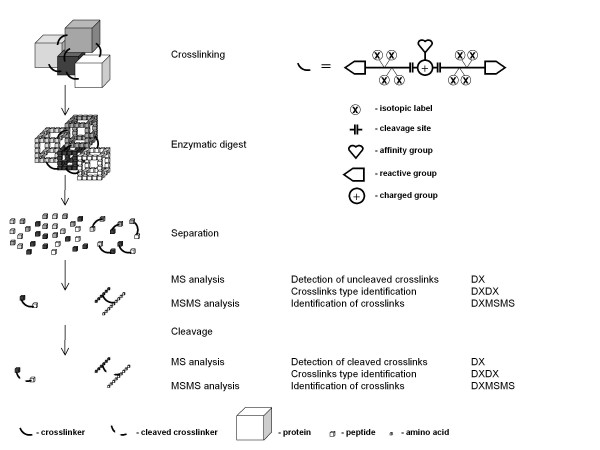
**Scheme of a typical crosslinking experiment, showing the corresponding steps of data analysis and appropriate programs**. Top right, scheme for an isotopically-coded cleavable crosslinker reagent possessing optional affinity and charge groups.

The overall structure of the ICC-CLASS program is similar to the general workflow of a typical experiment using isotopically-coded cleavable crosslinking reagents (Figure 1). By crosslinking a protein or a protein complex with an equimolar mixture of light and heavy forms of the crosslinking reagents, followed by enzymatic digestion of the crosslinked proteins, peptide crosslinks are obtained. These crosslinks, which may be chromatographically separated or affinity enriched, are then analyzed by MS and MS/MS. At this point, the MS spectra can be searched for the presence of specific isotopic signatures for *uncleaved *crosslinks using the DX program. Any crosslinks detected can be subsequently be targeted for MS/MS analysis, and the uncleaved crosslinks can be tentatively assigned using the DXMSMS program. Following cleavage of the crosslinker, the MS spectra can be searched again with the DX program, but this time searching for isotopic signatures specific to *cleaved *crosslinks.

At this step, the cleaved and uncleaved crosslinks can be matched to each other using the DXDX program, thus identifying dead-end, intra-peptide crosslinks, as well as possible cleaved components of the inter-peptides crosslinks. A dead-end crosslink is a single peptide modified with only one reactive group of the crosslinker reagent, while the second group is hydrolyzed or blocked. An intra-peptide crosslink is a single peptide where two residues within the same peptide are crosslinked to each other. An inter-peptide crosslink is a pair of peptides bridged with a crosslinker molecule. The cleaved DX mass list can be used again as an additional restricting input parameter for the DXMSMS assignments of the uncleaved crosslinks (see below). Finally, the assignments can be verified by MS/MS analysis of the cleaved crosslinks -- by matching their fragment ion masses with predicted masses from DXMSMS.

The ICC-CLASS software package thus provides a means of automating every data analysis step in a mass spectrometric experiment done with isotopically-coded cleavable crosslinkers. This software facilitates the assignment of these crosslinks, while the use of cleavable crosslinkers strengthens the confidence of these assignments.

### Searching for doublets (DX)

The search for doublets of peaks is based on sequential differential searching of the mass list for pairs of masses with a specified difference which corresponds to the mass difference between the heavy and light isotopic forms of the specific crosslinking reagent used (Table 1). The DX module is available as stand-alone Windows application as well as a macro for use with AB's Data Explorer software. Input parameters include a DX value for the mass difference due to the isotopic coding, a tolerance value for the experimentally-achievable precision (which is instrument dependent), and a minimum peak intensity cut-off (in case of the macro for Data Explorer). The most common DX mass differences can be pre-selected, but customized mass difference input values can also be used. The input mass lists should be stored as single columns of values in text files and thus are application and instrument independent. Mass lists of multiple spectra can be analyzed together. The results are stored in two text files: 1) the "total DX mass list file", which contains the masses of any doublets found, the residual values (*i.e*., the difference between the theoretical and observed doublet mass shift), and the names of the original individual mass list files for each spectrum; and 2) the "total mass list file" containing all of the masses, and the file names of the corresponding mass spectra.

**Table 1:** Isotopic coding mass differences.

Coding	N	H (1.00728 Da)	D (2.01355 Da)	D-H (+1)	D-H (+2)	D-H (+3)
H12/D12	12	12.08736	24.1626	12.07524	6.03762	4.02508
H8/D8	8	8.05824	16.1084	8.05016	4.02508	2.68339
H4/D4	4	4.02912	8.0542	4.02508	2.01254	1.34169

Isotopic coding is achieved by using light and heavy forms of the crosslinking reagents. Reaction products from an equimolar mixture of isotopically coded reagents will appear in the mass spectra as doublets of peaks of equal intensity, corresponding to the light and heavy forms of the reagents, separated by a specific mass difference resulting from the mass difference between the different forms of the reagent, and the charge state of the crosslinked ions (+1, +2, +3 are shown).

### Comparison of the uncleaved and cleaved doublets (DXDX)

**Figure 2:**
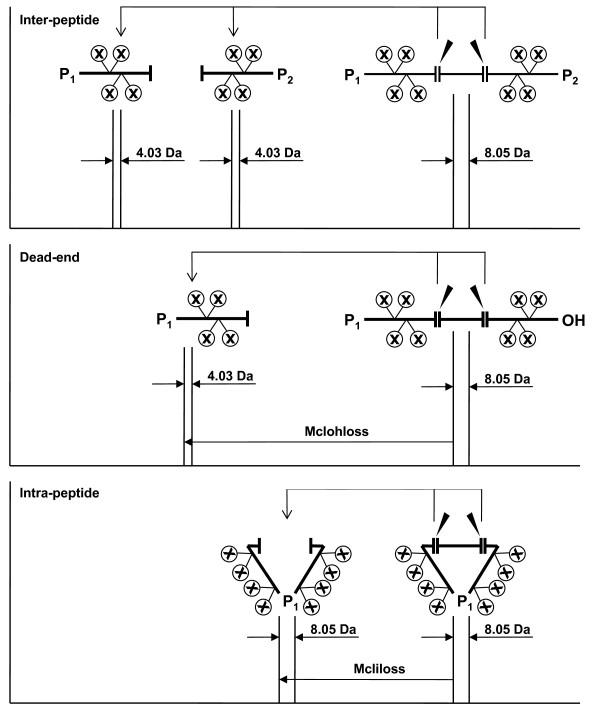
**Scheme of cleavage of inter-peptide (top), dead-end (middle), and intra-peptide (bottom) crosslinks**. An example with eight deuterium atoms as isotopic labels is shown. Dead-end and intra-peptide crosslinks can be identified based on specific mass losses (Mclohloss and Mcliloss, respectively), due to cleavage of the crosslinker.

**Figure 3:**
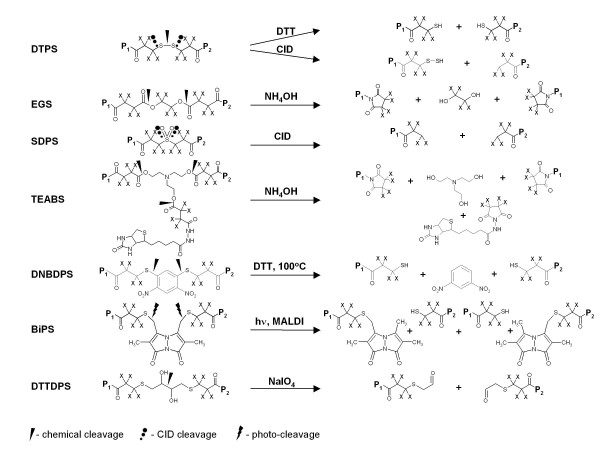
**Examples of isotopically-coded cleavable reagents and corresponding cleavage products**. X denotes hydrogen or deuterium for the light and heavy forms of the reagents, respectively.

The combination of isotopic coding with cleavage of the crosslinker creates a unique opportunity for identification of the crosslink type: dead-end, intra-, and inter-peptide. Importantly, cleavage of the crosslinker results in cleavage products with different mass changes and different isotopic coding for these three types of crosslinks (Figure 2). Thus, for example, in case of DTSP-H8/D8 (Figure 3), a crosslinker with eight aliphatic hydrogen/deuterium atoms in the spacer, cleavage with DTT will transform an uncleaved dead-end crosslink doublet of peaks 8.05 Da apart into cleaved crosslink doublet of peaks 4.03 Da apart, 104 Da lower in mass. An intra-peptide crosslink using DTSP-H8/D8, after cleavage, will appear in the mass spectrum as a doublet of peaks 8.05 Da apart with a gain of mass of 2 Da due to reduction of the disulfide bond and incorporation of two H atoms. The knowledge of the exact cleavage reaction chemistry for each crosslinking reagent is critical for establishing correct mass relationships between uncleaved and cleaved crosslink masses. These masses and the mass relationships between uncleaved and cleaved crosslinks can be conveniently used for rapid discrimination of the crosslink types. This allows one to focus the downstream analysis on the most structurally-informative inter-peptide crosslinks.

To automate the process of crosslink-type identification, we have developed a program which we call DXDX. This program searches for doublets of peaks corresponding to uncleaved and cleaved crosslinks, and then looks for pairs of uncleaved and cleaved doublets which differ in mass by values characteristic for each crosslink type, the type of cleavage reaction, and the crosslinking reagent used (Table 2). These masses can be calculated based on precise knowledge of the chemistry of the cleavage reactions (Figure 3). The DXDX program can process multiple pairs of uncleaved and cleaved spectra at once. The input mass lists from the corresponding uncleaved and cleaved experiments should be stored in two text files each containing two columns of mass values and the corresponding spectrum designations. The "total mass list" output file from the DX program can be used as the input file for the DXDX program. The order of the corresponding pairs of mass spectra in these uncleaved and cleaved mass list files must be the same. Control values include a choice of the most commonly-used crosslinkers, as well as user-definable values for the mass relationships between uncleaved and cleaved crosslinks. The output includes a list of the doublet pairs for dead-end, intra-, and inter-peptide crosslinks found. The mass differences between the observed and calculated masses (*i.e*., the mass residuals) are also given for each uncleaved-cleaved crosslink pair.

**Table 2:** Mass additions for crosslinks cleavage products.

Reagent	Cleavable	Mclrest	Mclirest	Mcliploss	Mclohloss	Mcliloss
DTSP	DTT	87.99829	175.99657	-3.02349	103.99320	-2.01566
	CID	119.96981	-	-1.00727	72.02058	-
		54.01002	-	-1.00727	137.98037	-
EGS	NH_4_OH	82.00548	164.01096	61.02895	162.05282	62.03678
BiPS	hν	278.07251	173.98091	-3.02349	103.99320	190.07422
		87.99829	173.98091	-3.02349	294.06743	190.07422
DNBDPS	DTT, 100°C	87.99829	175.99657	162.97852	269.99465	163.98581
TEABS	NH_4_OH	82.00548	164.01096	488.21844	589.24121	489.22571

The masses of the cleaved crosslinks can be calculated using following formulas:

[M_12_+H]^+ ^= [M_1_cl+H]^+ ^+ [M_2_cl+H]^+ ^+Mcliploss

[M_1_OH+H]^+ ^= [M_1_cl+H]^+ ^+Mclohloss

[M_1_i+H]^+ ^= [M_1_icl+H]^+ ^+Mcliloss

[M_1_cl+H]^+ ^= [M_1_+H]^+ ^+Mclrest

[M_1_icl+H]^+ ^= [M_1_+H]^+ ^+Mclirest

where H -- mass of proton; M_1_, M_2 _-- masses of free peptides; M_12 _-- mass of inter-peptide crosslink; M_1_OH -- mass of dead-end crosslink; M_1_i -- mass of intra-peptide crosslink; M_1_cl -- mass of cleaved dead-end or inter-peptide crosslink; M_1_icl -- mass of cleaved intra-peptide crosslink; Mcliploss, Mclohloss, Mcliloss -- mass additions for cleaved inter-peptide, dead-end and intra-peptide crosslinks, respectively; Mclrest, Mclirest -- mass of cleaved portion of the crosslinking reagent for cleaved inter-peptide or dead-end and intra-peptide crosslinks, respectively.

### Crosslink identification based on MS/MS fragmentation data (DXMSMS)

The cornerstone of successful crosslinking applications in structural proteomics is the confident identification of the crosslinks. Unfortunately, even if one cleaves the crosslinks and uses high mass-accuracy instruments, it is still challenging to unambiguously identify crosslinks based solely on mass, especially in digests from complex protein systems. MS/MS fragmentation information on the crosslinks needs to be included in the analysis in order to provide confirmation of the identification. MS/MS fragmentation of the individual peptides obtained by cleavage of the inter-peptide crosslinks provides an additional level of confidence in the identifications by providing partial sequence information for the peptides forming the crosslink. To address the complex nature of the MS/MS spectra from inter-peptide crosslinks, we incorporated additional features into the analysis of the fragmentation data by the DXMSMS program. Fragmentation of the isotopically-coded crosslinks produces two types of ions: ions which contain the isotopically-coded crosslinker, and ions that are not isotopically coded. Isotopically-coded fragment ions are the most informative and important for confident assignment of the crosslinks. Distinguishing between these two types of ions provides additional specificity for assignment of the crosslink fragments. Finally, the fragmentation of the uncleaved crosslink is compared with the fragmentation of from the cleaved crosslinks. If corresponding ions are observed in both spectra, this provides additional confirmation of the crosslink assignment. Thus, we have designed our DXMSMS program to analyze MS/MS fragmentation data of the crosslinks by incorporating separate inputs for isotopically-coded and non-coded fragment ions from uncleaved crosslinks. In addition, the masses of the predicted fragments from the cleaved crosslinks are included in the output.

The algorithm for assignment of the crosslinks is based on searching for a given mass within all possible peptide combinations for the protein sequences given and the crosslinking reagent used (Table 3). The number of possible choices is then reduced by several optional additional restrictions. These include the presence of isotopically uncoded and/or coded MS/MS fragments, possible cleavage products, the presence of a specific peptide subsequence deduced from *de novo *sequence analysis of the MS/MS spectra, the absence of certain amino acids in the crosslink, the presence of aspartic acid at the site of fragmentation (which usually produces the most intense isotopically-coded fragment due to facile fragmentation [15]), the possible crosslinking and enzymatic digestion sites, and the type of crosslink.

**Table 3:** Mass additions for crosslinking reaction products.

	Mip	MOH	Mi	MNH_2_
DSS	137.06025	156.07864	138.06808	155.09462
DTSP	172.97310	191.99149	173.98093	191.00747
EGS	225.03991	244.05830	226.04774	243.07428
BiPS	363.04732	382.06571	364.05515	381.08169
DNBDPS	338.97455	357.99294	339.98238	357.00892
TEABS	652.22885	671.24724	653.23668	670.26322

The masses of the crosslinked products for the light form of the reagents can be calculated using following formulas:

[M_12_+H]^+ ^= [M_1_+H]^+ ^+ [M_2_+H]^+ ^+Mip

[M_1_OH+H]^+ ^= [M_1_+H]^+ ^+MOH

[M_1_i+H]^+ ^= [M_1_+H]^+ ^+Mi

[M_1_NH_2_+H]^+ ^= [M_1_+H]^+ ^+MNH_2_

where H -- mass of proton; M_1_, M_2 _-- masses of free peptides; M_12 _-- mass of inter-peptide crosslink; M_1_OH -- mass of dead-end crosslink; M_1_i -- mass of intra-peptide crosslink; M_1_NH_2 _-- mass of dead-end amide crosslink (if the reaction was quenched with ammonium salts); Mip, MOH, Mi -- mass additions for inter-peptide, dead-end, and intra-peptide crosslinks, respectively.

The output for every match includes 1) the theoretical mass of the crosslink, 2) the mass differences between the experimental and theoretical masses in ppm, 3) the sequence of the crosslink, and 4) the masses of all possible b- and y- ions for each intact and crosslinked peptide, 5) the masses of the crosslink cleavage products, and 6) the fragment ion masses for the individual cleaved peptides in the case of cleavable crosslinkers. This information allows to one to re-inspect the MS/MS spectra and make confident crosslink assignments.

### Applications of the ICC-CLASS software suite

**Figure 4:**
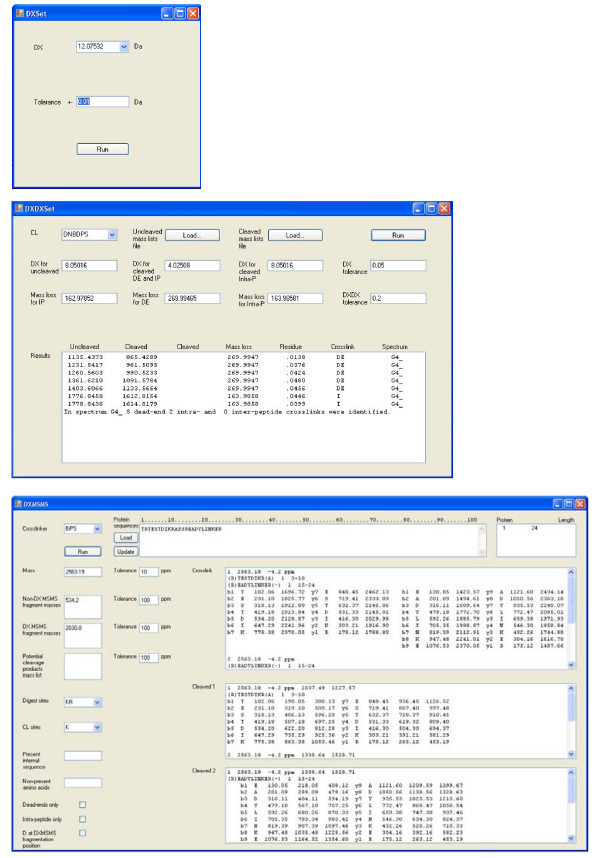
**Graphic user interfaces of the DX, DXDX, and DXMSMS programs**.

The ICC-CLASS software suite has been used for the analysis of numerous protein crosslinking experiments with LC/MALDI-MS, utilizing several isotopically-coded cleavable crosslinking reagents [4,16] which are available at http://www.creativemolecules.com (Figure 3). Graphic user interfaces and examples of the output from these programs are presented in Figure 4.

A typical crosslinking experiment is done by offline LC separation followed by MALDI analysis of the HPLC fractions. For a mid-size protein complex, 50-100 kDa, this produces one to two hundred crosslinked peptide species, which are usually distributed over 24 to 48 fractions from a reversed-phase HPLC gradient. Following acquisition of the MS spectra, the mass lists for all of the fractions are searched with DX for doublets of signals corresponding to the isotopic coding of the crosslinker used. An important parameter for this step is an appropriate signal cut-off value to eliminate false-positive signals derived from noise. For an AB 4800 TOF/TOF instrument, we normally start with a value of 50 counts for this parameter. A strict tolerance value for the doublets mass difference is also helpful. We normally use values of 0.01 to 0.05 Da. Using DXDX for screening spectra from corresponding uncleaved and cleaved fractions allows one to rapidly eliminate most of the dominant (and less-informative) dead-end and intra-peptide crosslinks from downstream analysis, and to more easily identify potential inter-peptide crosslinks which are more structurally informative. At this point, the mass lists containing the masses of the potential cleaved and uncleaved inter-peptide crosslinks can be converted into an inclusion list for automated MS/MS acquisition. These MS/MS spectra can be subsequently analyzed with DXMSMS.

Several considerations which are intrinsic to crosslinking studies need to be taken into account. In the case of the analysis of a fixed number of chromatographic fractions, the complexity of the spectra will inevitably increase with increasing complexity of the system studied. The probability of detecting uncleaved-cleaved pairs of false doublets which satisfy the mass relationships of the cleavage reaction is negligibly low. However, eventually, increasing sample complexity could potentially lead to overlap of signals from uncleaved and cleaved crosslinks, with signals from free peptides, and to potentially increasing the chances of detecting "false doublets" in the spectra. This could interfere with fully-automated crosslink identification. This, however, is a common complication of crosslinking applications in complex protein systems. There are two possible solutions to this problem - the first is to reduce the complexity of the mixture by collecting additional fractions or by adding by additional stages of separation, *e.g*., multidimensional chromatography. The second alternative is to use affinity-purifyable isotopically-coded cleavable crosslinkers (Petrotchenko E. V., Thomas J. M., Borchers C. H.: DNBDPS: an isotopically-coded cleavable crosslinker affinity-purifyable with antibodies, submitted; Petrotchenko E.V., Serpa J. J., Borchers C. H.: An Isotopically-coded CID-cleavable biotinylated crosslinker: CBDPS, submitted). Affinity enrichment of the crosslinks eliminates most of the interfering signals due to non-crosslinked peptides and simplifies and clarifies the spectra. This makes the matching the un-cleaved and cleaved crosslinks masses, as well as acquisition of the MS/MS spectra, more feasible for complex protein systems. The situation where the mass of one of the cleaved products of an inter-peptide crosslink falls below the usual range for MALDI-MS (< 800 Da) also needs to be considered. In this case, the uncleaved crosslink will not be identified as inter-peptide by DXDX, but post-analysis re-acquisition of the MS spectra over a lower molecular weight range can often resolve this issue.

**Figure 5:**
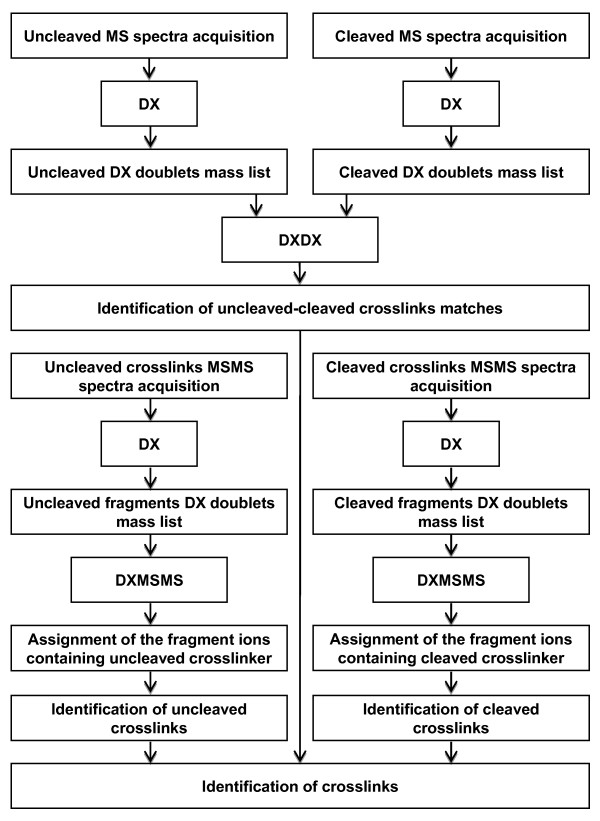
**Flowchart for a crosslinking experiment with ICC-CLASS data analysis**.

A workflow diagram of data analysis using ICC-CLASS is presented in Figure 5. Using this software package allows one to complete an entire experiment, including data analysis, in a matter of several days compared to several weeks if one tried to analyze the data manually. Using isotopically-coded crosslinking reagents, together with ICC-CLASS, dramatically speeds-up the analysis and raises the confidence of crosslink assignments. Further developments may include adapting the software for the automated acquisition/analysis of LC/ESI-MS/MS data for isotopically-coded CID-cleavable crosslinking reagents and generating a scoring function for pre-filtering the final crosslinks assignments.

## Conclusions

ICC-CLASS is a collection of programs which greatly facilitates data analysis from experiments using isotopically-coded cleavable crosslinking reagents. Together with advanced crosslinking reagents, selective crosslink purification strategies, and sophisticated mass spectrometric techniques, it provides a powerful analytical toolkit for structural proteomics crosslinking applications.

## Availability and requirements

**Project name: **ICC-CLASS (DX, DXDX, DXMSMS).

**Project home page: **http://www.proteincentre.com/services/structural-proteomics, http://www.creativemolecules.com/CM_Software.htm

**Operating system: **Windows XP.

**Programming language: **Visual Basic.

**Other requirements: **.NET Framework.

**License: **not required.

**Restriction to use by non-academics: **none.

## Abbreviations

MALDI-MS: matrix-assisted laser desorption/ionization mass spectrometry; LC/MS: liquid chromatography with mass spectrometry detection; MS/MS: tandem mass spectrometry; ESI- MS: electrospray ionization mass spectrometry; HPLC: high performance liquid chromatography; CID: collision-induced dissociation.

## Authors' contributions

EP designed the algorithms, wrote the programs and performed the analyses. CB oversaw the project. Both authors read and approved the final manuscript.
